# The impact of Medicare part D on income-related inequality in pharmaceutical expenditure

**DOI:** 10.1186/s12939-019-0955-9

**Published:** 2019-04-16

**Authors:** Natalie Carvalho, Dennis Petrie, Linkun Chen, Joshua A. Salomon, Philip Clarke

**Affiliations:** 1 Centre for Health Policy, Melbourne School of Population and Global Health, The University of Melbourne, Level 4, 207 Bouverie St, Carlton, VIC 3010 Australia; 2 Centre for Health Economics, Monash Business School, Monash University, Building H, Level 5, Caulfield, VIC 3145 Australia; 3 Department of Global Health and Population, Harvard T.H. Chan School of Public Health, 655 Huntington Avenue, Boston, MA 02115 USA

**Keywords:** Medicare part D, Inequality, Concentration index, Health insurance, prescription drugs

## Abstract

**Background:**

Income-related inequality measures such as the concentration index are often used to describe the unequal distribution of health, health care access, or expenditure in a single measure. This study demonstrates the use of such measures to evaluate the distributional impact of changes in health insurance coverage. We use the example of Medicare Part D in the United States, which increased access to prescription medications for Medicare beneficiaries from 2006.

**Methods:**

Using pooled cross-sectional samples from the Medical Expenditure Panel Survey for 1997–2011, we estimated income-related inequality in drug expenditures over time using the concentration and generalised concentration indices. A difference-in-differences analysis investigated the change in inequality in drug expenditures, as measured using the concentration index and generalised concentration index, between the elderly (over 65 years) and near-elderly (54–63 years) pre- and post-implementation of Medicare Part D.

**Results:**

Medicare Part D increased public drug expenditure while out-of-pocket and private spending fell. Public drug expenditures favoured the poor during all study periods, but the degree of pro-poorness declined in the years immediately following the implementation of Part D, with the poor gaining less than the rich in both relative and absolute terms. Part D also appeared to result in a fall in the pro-richness of private insurance drug expenditure in absolute terms but have minimal distributional impact on out-of-pocket expenditure. These effects appeared to be short lived, with a return to the prevailing trends in both concentration and generalised concentration indices several years following the start of Part D.

**Conclusions:**

The implementation of Medicare Part D significantly reduced the degree of pro-poorness in public drug expenditure. The poor gained less of the increased public drug expenditure than the rich in both relative and absolute terms. This study demonstrates how income-related inequality measures can be used to estimate the impact of health system changes on inequalities in health expenditure and provides a guide for future evaluations.

**Electronic supplementary material:**

The online version of this article (10.1186/s12939-019-0955-9) contains supplementary material, which is available to authorized users.

## Background

Income-related inequality measures such as the concentration index are often used to describe the unequal distribution of health and health care access in a single measure. Measuring the inequality in use and expenditure on health services has received considerable attention in recent years. Studies have assessed income-related inequality in medical care expenditures at a point in time in the United States (U.S.) [1] and other Organisation for Economic Co-operation and Development (OECD) countries [2–4]. A smaller number of studies have provided descriptive analyses of changes in access to care over time [5–7], or changes in inequality of self-reported health before and after a major health system reform [8]. However, to our knowledge, there have been few cases where inequality measures have been used to evaluate the impact of a policy-wide change on access to care or health expenditure. This study takes that step, demonstrating the use of such measures to evaluate changes in the degree of progressivity in health care expenditures as a result of implementation of the Medicare prescription drug benefit (Part D) in the U.S.

### Medicare part D prescription drug program

Medicare is a national social insurance program covering 46 million adults aged 65 years or older and 9 million younger adults with permanent disabilities [9]. Nearly half (45%) of beneficiaries have four or more chronic conditions and one in four (26%) report being in fair or poor health [10]. The majority of Medicare beneficiaries take at least one medication [11]. Prior to 2006, Medicare only covered prescription drugs administered in a physician’s office or institutional setting. The Medicare Modernization Act of 2003 provided the legislation that enabled the Medicare prescription drug benefit plan (Part D) to be implemented, on January 1, 2006, allowing Medicare beneficiaries to voluntarily access subsidized outpatient prescription drug coverage offered through stand-alone prescription drug plans and Medicare Advantage prescription drug plans. Prior to Part D, 48% of Medicare beneficiaries already had relatively generous drug coverage through their former employer (34%) or Medicaid (14%), one third had more limited coverage through privately purchased Medigap or Medicare Advantage plans, and 18% had no coverage [11]. Despite some initial difficulties in Part D uptake in early 2006, and although enrolment was voluntary for Medicare-eligible citizens not dually enrolled in Medicaid [12], by 2015, an estimated 42 million Medicare beneficiaries were enrolled in Medicare drug plans, representing about 76% of all eligible beneficiaries [10].

While the available drug plans varied considerably across the country, all drug plans are required to offer a benefit that is actuarially equivalent or more generous to a defined “standard” prescription drug benefit [10]. However, monthly premiums and cost-sharing amounts for drugs vary widely across plans and regions of the country, as do the list of covered drugs included within plans’ formularies. For instance, the average monthly Part D premium in 2015 for prescription drug plans was $38.83, but ranged from $12.60 to $171.90 [10]. Through the Low-Income Subsidy (LIS) program 30 % of Part D enrolees (11.5 million beneficiaries) receive additional premium and cost-sharing assistance [10]. Beneficiaries who are dually eligible for Medicare and Medicaid are enrolled in plans with no premiums or deductable under Part D, which replaces their previous coverage through Medicaid [13].

Total public drug spending increased from $48.1 billion in 2006 to $67.4 billion in 2012 [14]. The Congressional Budget Office estimates that Part D spending will total $76 billion in 2015, representing 14% of total Medicare spending, with this proportion expected to rise to 17% by 2023 [15]. In light of the rising costs of Medicare on the federal budget, the sustainability of the program has been questioned [16].

Many prior studies have assessed the impact of Part D on Medicare beneficiaries’ drug use, expenditures and as well as distributional effects. These studies have found that Part D increased drug use among the elderly on average by 6–16% [12, 17–19], was associated with reductions in Medicare beneficiaries’ out-of-pocket (OOP) drug costs [13, 17–23], particularly at the highest end of the expenditure distribution [12], but those with the highest drug spending still had substantial OOP costs [17]. Other studies examined the impacts of Part D on total and OOP drug expenditures for different ethnic groups in the US [21, 24, 25], finding mixed results on whether ethnic groups such as African Americans and Hispanics experienced a greater reduction in annual total and OOP drug expenditures than whites. Interestingly, one study found a reduction in prescription drug prices as a result of Part D [26].

Most studies focus on total and OOP drug expenditures, without looking specifically at drug expenditures from public sources. However, prescription drug expenditures account for an important and growing component of public expenditures. While all of these studies have provided valuable insights into the association between Part D and drug expenditures, to our knowledge no study has examined how Part D changed the degree of income-related inequality of prescription drug expenditures. With the objective of improving access to prescription drugs, Part D is likely to have important implications for equity, particularly given the existing disparities in access to medical care [19].

## Methods

### Income-related inequality measures

The concentration index (CI) and generalised concentration index (GCI) are used to estimate relative and absolute income-related inequality in drug expenditures respectively [27]. Both are derived from the concentration curve, which plots the cumulative proportion of drug expenditures against the population ranked by income [27, 28]. The CI is twice the area between the drug expenditure concentration curve and the line of equality [28], and ranges from − 1 to 1. If expenditures are concentrated among the poor (“pro-poor”), the concentration curve falls above the 45-degree line of equality, and CI < 0. CI is 0 when the concentration curve coincides with the diagonal. The GCI is simply the CI multiplied by the mean expenditure [27, 29].

While the CI measures *relative* income-related inequality in expenditures; inequality preserved by a proportional increase in health care expenditures for all, the GCI measures *absolute* inequality; inequality preserved by an equal absolute increase for all [30–32]. Deriving both the CI and GCI allows the nature of the inequality in health expenditure changes to be more fully considered.

We standardise expenditure for need using age and self-reported health to remove the effect of these factors on income-related inequality in drug expenditure [33]. As a robustness check, we also standardise using the presence of diabetes, hypertension, mental health disorders, and cancer. We consider both direct and indirect standardisation techniques [2, 33–36] – see Additional file 1 for details. To further understand those factors associated any change in the CI of public drug expenditure, we decompose the directly standardised partial CI into the policy relevant variables including income, gender and ethnicity, and report on the share of public drug expenditure CI explained by each of these variables.

### Data

We use data from the Medical Expenditure Panel Survey (MEPS) from 1997 to 2011. MEPS is a nationally representative survey of the non-institutionalised, civilian population in the U.S. [37] and involves an overlapping panel design in which two calendar years’ of household and individual-level information is collected through a series of five rounds of interviews over a two-and-half year period. The dataset contains household and personal demographic information as well as individuals’ heath care expenditure and health insurance coverage in each year. Data on prescription drugs were obtained from survey participants, with permission sought to collect more detailed information (type, dosage and payment for each filled prescription) from pharmacy records. While previous research has found some underreporting of the number of drugs taken by households, the degree of underreporting was consistent across socioeconomic groups, and validation with Part D claims data showed good accuracy of overall number of drug fills and total drug expenditures [38]. MEPS does not include payments for over-the-counter medications or prescription drugs obtained in a physician’s office, during hospital visits or in other institutional settings.

We use the Full-Year Consolidated Data files for household and personal demographic information, such as age, sex, race or ethnicity, and drug expenditures. Total drug expenditures are separated into sources including OOP, public and private insurance. Public insurance includes payments made through Medicaid, Medicare, Veterans’ Administration, other Federal, State and Local sources, and Worker’s Compensation. Medicare payments alone are not considered separately from all public expenditure. Payments from unclassified or unknown sources are excluded [37]. Part D premiums are not available in MEPS so were excluded in the analysis apart from robustness checks using imputed premiums for low-income beneficiaries. The Medical Conditions files were used to identify individuals with diabetes, hypertension, mental health disorders and cancers based on self-report.

### Sample and analysis

Because inequality measures often require large sample sizes to obtain robust results, we pool three years of data together starting from 1997 to construct pooled cross-sectional samples over time. We therefore obtain three sets of observations before Part D was implemented (1997–99, 2000–02, 2003–05) and two sets of observations after Part D went into effect (2006–08, 2009–11).

We restrict our sample to Medicare beneficiaries aged 65 years or older as our treatment group. As others have done, we form a control group made up of non-elderly [13, 19, 20, 22, 39]. Given the nature of our analysis in which we use concentration indices to describe inequality in the population, we include a fairly wide age range for the non-elderly control group (aged 54 to 63 years), in line with other studies [20, 39]. In order to assess the impact of Part D, we performed a difference-in-differences (DID) estimation [40] to investigate whether the changes in inequality of drug expenditure from 2003 to 05 to 2006–08 were significantly different between those aged 54–63 and those 65 years and over. A DID design has been used previously in prior evaluations of the impact of Part D [12, 19, 39]. The one-year gap between the control and treatment groups is to ensure the samples do not overlap with each other due to the overlapping panel design within MEPS [13]. Also, the implementation of Part D may have reduced expenditure for those close to the eligibility threshold who may have delayed the uptake of prescriptions until they became eligible for Medicare. As used by others who have evaluated Part D [12, 19, 39], a DID approach controls for time-invariant factors within the groups and time-varying factors on the inequality of prescription drug expenditure that were common for both under and over 65 s.

The DID estimator for a mean outcome is commonly estimated within a regression framework where additional individual controls can be directly included in the regression for the outcome (see Additional file 1 for further details). However, it is not possible to implement this regression DID framework for inequality indices, so instead we present a simplified DID estimation framework.

The DID estimator can be expressed as:

$$ \delta =\left({y}_{I, post}-{y}_{I, pre}\right)-\left({y}_{C, post}-{y}_{C, pre}\right) $$

Where *δ* is the DID estimator; y is the outcome of interest (as explained below); *I* indicates the intervention group; *C* indicates the control group; post is the period from *2006 to 08* following the policy change; pre- is the period from *2003 to 05* before the policy change. No additional controls were included, however controls are implicitly included during the prior standardization process, as explained previously (standardizing for need using age and self-reported health), with additional standardization controls tested in robustness checks (Additional file 1).

Three separate DID estimations were calculated. In the first, y is mean drug expenditure (across all expenditure sources and then split out by source of expenditure). In the second, y is the CI of drug expenditure (again presented for total expenditure, and split out by source). Finally in the last estimation, y is the GCI of drug expenditure, similarly presented for total expenditure and by payer category (Table 2).

We use real equivalized household income using the OECD-modified equivalence scale [41] to rank individuals (as used in similar studies eg. Van Doorslaer and Masseria, 2004; Cabieses et al., 2015), which is is the standard approach used in the income inequality literature. This scale, which allows households in a population to be assigned a value in proportion to its needs, assigns a value of 1 to the household head, 0.5 to each additional adult member, and 0.3 to each child. The family unit was identified using the MEPS definition (see Additional file 1) and expenditures were adjusted to 2012 dollars using Consumer Price Index (CPI) deflators [42].

To standardise drug expenditure for need, we define dummy variables for each five-year age group and one that covers those aged 80 or over. We also define four categories for self-reported health: excellent, very good, good, fair or poor. In the case of different levels of self-reported health within the same survey year, we use the worst self-reported health.

We use bootstrapping with 1000 replications to explore the statistical significance of our results, with re-sampling at the individual level to take individual clustering into account. The MEPS person-level weights were used for all analyses, and adjust for the complex sampling design. All analyses were carried out using Stata version 14.0.

### Robustness checks

We carry out several robustness checks to explore the impact of key assumptions and data limitations on our findings. First, we include additional indicators for four conditions associated with increased prescription drug use to further standardise expenditures for need. We identify individuals with relevant medical conditions using the following 3-digit International Classification of Diseases, Ninth Revision, Clinical Modification (ICD-9-CM) codes: 250 (diabetes), 401 (hypertension), 290–319 (mental health disorders), and 140–239 (cancers). Next, we carry out a sensitivity analysis aimed at capturing public expenditure related to assistance with Part D premiums for LIS-eligible beneficiaries. While cost-sharing subsidies are already captured within drug expenditures, our analysis does not include spending on drug plan premiums. We add in national average premium subsidies for dual eligible beneficiaries (those eligible for both Medicaid and Medicare) and those below the 100% of the Federal Poverty Line (FPL), based on data from the Kaiser Family Foundation [43]. National average monthly premiums (adjusted to 2012 dollars) varied by year (ranging from $25.93 in 2006 (unadjusted to 2012 dollars), to $37.78 in 2012), and were based on stand-alone prescription drug plans (PDPs) since the majority of Part D enrollees receiving LIS are enrolled in stand-alone PDPs [43]. These amounts were added first as an income effect (which impact on income rankings within the population) and second as if the full amount of premium assistance was spent on prescription medications (assigned to public sources of expenditure), in order to get a lower- and upper-bound on the effect of premium subsidies. Next, we rerun the analysis excluding individuals covered by Medicare in the control group (54–63 years). Third, we re-estimate the DID results without the 2006 and 2007 data, using 2008–2010 as the post-implementation period. This does not capture the immediate impact following the policy change, but estimates the policy impact after a few years of implementation where some initial difficulties in Part D uptake in early 2006 [12] are no longer present and more generally allows for a period of individual adjustment to the changed policy environment, as well as excluding potential misclassification errors of private drug expenditures as public expenditure in the 2007 MEPS data [44].

## Results

All individuals in our sample have full information on age, income, gender and ethnicity. We exclude approximately 0.3% of individuals in each year who have missing data on self-reported health. The final sample sizes for the descriptive statistics range from 6869 in 1997–99 to 11,485 in 2009–11 for the under 65 s, and from 9516 in 1997–99 to 11,817 in 2009–11 for the over 65 s. For the DID analysis, the final sample sizes range from 9588 in 2003–05 to 10,237 in 2006–08 for the under 65 s, and from 11,225 in 2003–05 to 11,004 in 2006–08 for the over 65 s.

Table 1 provides weighted descriptive statistics of the Medicare eligible elderly population in each pooled sample period. Males represented 42–44% of the over 65 s. Around 80% in each period were white, 8% black and 5–7% Hispanic. Following the implementation of Medicare Part D, 54.5% of the over 65 s reported having Medicare Part D coverage in 2006–08. This percentage increased to 61% in 2009–11. The proportion reporting Medicare Part D coverage was negatively associated with income group (see Additional file 1: Figure S6).

**Table 1:** Descriptive statistics for the over 65 s

	1997–99	2000–02	2003–05	2006–08	2009–11
Percentage of sample					
Male	42.1	42.3	42.9	42.9	43.7
Ethnicity					
White	84.3	82.8	81.1	79.7	79.3
Black	8.29	8.34	8.28	8.54	8.53
Hispanic	5.18	5.67	6.23	6.96	7.22
Other	2.22	3.24	4.42	4.80	4.91
Medicare Part D Coverage	–	–	–	54.5	61.0
2012 US dollars					
Income (thousands)	34.4 (31.1)	34.0 (29.2)	35.6 (30.3)	37.9 (33.3)	39.1 (34.4)
Total drug expenditure	1151 (1514)	1569 (1987)	2144 (4037)	2287 (3340)	2275 (3678)
OOP	671 (1076)	865 (1278)	1092 (1617)	686 (1144)	525 (888)
Public	257 (714)	410 (1185)	565 (1533)	1263 (2427)	1299 (2883)
Private	223 (687)	295 (838)	486 (3356)	337 (1530)	450 (1520)
Observations	9516	11,102	11,225	11,004	11,817

Notes: Weighted statistics. Income and expenditure statistics are provided for mean and standard deviation, with the later in brackets. Income is equivalized household income

Total prescription drug expenditures increased over time. Expenditures covered by private insurance were reduced directly following Medicare Part D but later continued its upward trend. However, OOP payments and public insurance expenditures experienced dramatic changes in 2006–08, with a significant reduction in OOP costs and significant increase in public expenditures.

**Fig. 1:**
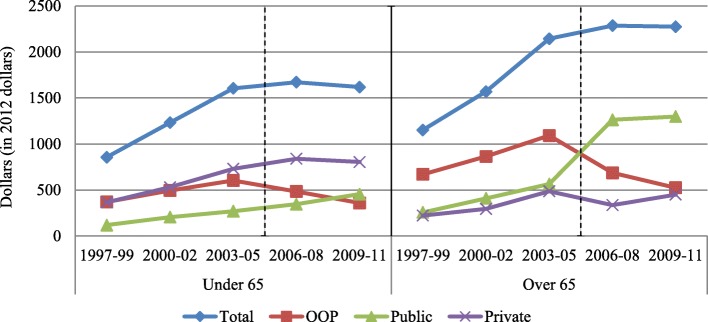
Weighted average drug expenditure per person. The dotted line indicates when Medicare Part D was implemented

Figure 1 shows average drug expenditures from all sources over time. Total drug expenditure was higher among the over 65 s over the entire period. For both groups, we observe an increasing trend in total expenditure from 1997 to 2005. Total drug expenditure remained stagnant over the period 2006–08 and 2009–11 in both the over 65 s ($2287 and $2275 respectively in each period) and the non-elderly group ($1671 and $1619 respectively in each period). Mean public drug expenditure increased over time in both groups. An increase in public drug expenditure was observed among the over 65 s following the implementation of Medicare Part D; the same was not seen among the under 65 s. On the other hand, OOP payments in both groups were increasing pre-Medicare Part D, and then declined from 2006 onwards.

**Fig. 2:**
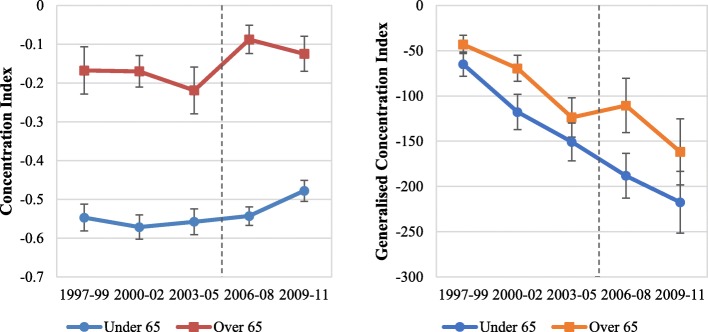
Income-related inequality in public drug expenditure. Weighted statistics. Left panel plots the CI of public drug expenditure in each period. Right panel plots the GCI of public drug expenditure in each period. Error bars represent the 95% confidence intervals for each estimated value. The dotted line indicates when Medicare Part D was implemented

Measures of inequality in total drug expenditures (CI and GCI) show that they were pro-poor (CI < 0 and GCI < 0) for both groups over all time periods (see Additional file 1: Figures S1 and S2). Public drug expenditure especially was pro-poor over all periods (Fig. 2), however the degree of pro-poor inequality was greater in the under 65 s (e.g., CI ~ − 0.55 prior to 2006) than the over 65 s (e.g., CI ~ − 0.2 prior to 2006). Inequality in OOP spending trended from more to less pro-poor over time. Inequality in private insurance drug payments were pro-rich for both groups across all time periods. Using direct or indirect standardised drug expenditure results in similar trends, however total and OOP expenditures became slightly pro-rich over time (see Additional file 1: Figures. S3 and S4). Focusing on public drug expenditures, the CI and GCI of public drug expenditure experienced a positive shock (indicating less pro-poorness in expenditure) for the over 65 s in the 2006–08 period; no similar trend was observed in the under 65 s (Fig. 2). For example, the CI in public drug spending for over 65 s rose from − 0.219 in 2003–2005 to − 0.088 in 2006–2008, while for under 65 s, the CI increased from − 0.558 to − 0.543 over the same period.

**Fig. 3:**
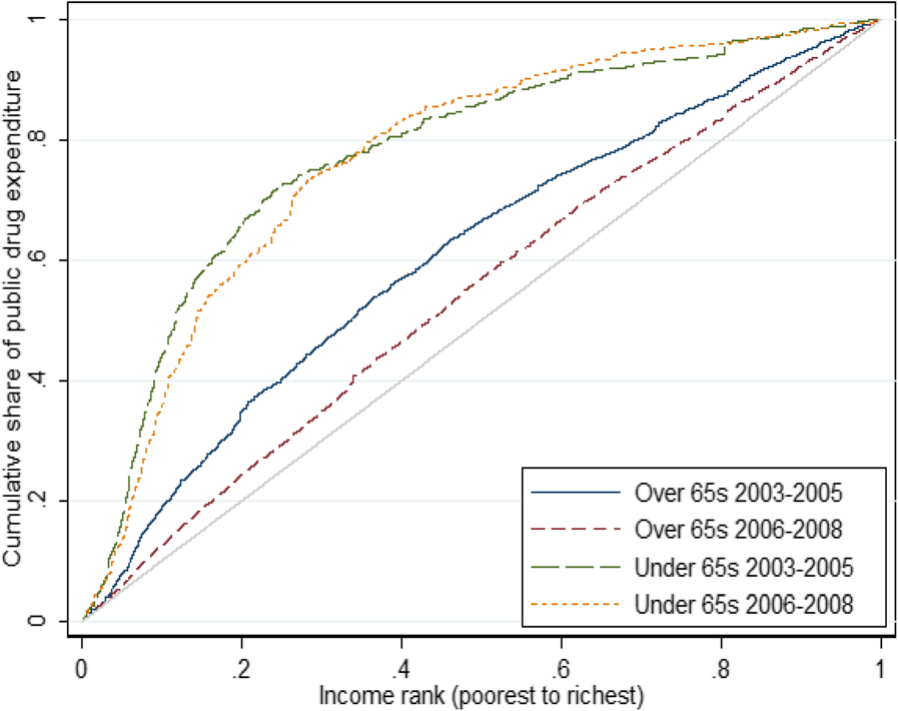
Concentration curve of public drug expenditure for under and over 65 s in the period pre-Medicare Part D (2003–05) and post-Medicare Part D (2006–08). The 45 degree line indicates line of perfect equality in public drug expenditures

To investigate where in the income distribution changes in public drug expenditure occur, Fig. 3 plots the concentration curve of public drug expenditure for both groups in 2003–05 and 2006–08. As expected, all concentration curves show pro-poor public drug expenditure. There is little difference in the two curves for the under 65 s. On the other hand, for the over 65 s, the concentration curve in 2006–08 shifted closer to the diagonal across the whole income distribution, compared to the 2003–05 period.

Table 2 shows the estimated DID coefficients of mean drug expenditure and the inequality measures of drug expenditure. From 2003 to 05 to 2006–08, OOP and private drug expenditures declined significantly while public drug expenditures significantly increased among the over 65 s as compared to those aged 54–63. There was a small and insignificant difference in total mean drug expenditure over time between groups. The DID estimates of CI and GCI in public drug expenditure were positive, both significant at 1%, indicating a significant impact of Part D on reducing the existing (pro-poor) inequality in public drug expenditures for the over 65 s. There were small and insignificant differences in relative inequality of OOP and private drug expenditure, and in absolute inequality of OOP costs between groups. In absolute terms, private drug expenditure decreased more among the over 65 s as compared to the under 65 s, with significant differences found for the standardised GCI.

**Table 2:** Difference-in-differences estimation in mean, CI and GCI of drug expenditure

	DID in mean drug expenditure	DID in drug expenditure CI			DID in drug expenditure GCI		
		Standardisation			Standardisation		
		None	Direct	Indirect	None	Direct	Indirect
Total	77.1 (0.820)	−0.007 (−0.247)	−0.010 (− 0.354)	−0.007 (− 0.271)	5.506 (0.107)	0.859 (0.017)	4.612 (0.098)
OOP	− 285.7^c^ (−7.541)	−0.005 (− 0.118)	0.010 (0.251)	0.011 (0.277)	3.738 (0.147)	7.063 (0.290)	6.018 (0.267)
Public	622.2^c^ (14.155)	0.117^b^ (2.810)	0.107^a^ (2.375)	0.107^a^ (2.573)	50.413^a^ (2.006)	68.255^b^ (2.999)	66.254^b^ (3.121)
Private	−259.4^c^ (−3.868)	0.040 (0.667)	0.030 (0.536)	0.027 (0.527)	−48.646 (−1.525)	−74.460^a^ (−2.177)	−67.659^a^ (− 2.192)

Notes: DID estimation obtained directly over the period 2003–05 and 2006–08. T-statistics, in brackets, based on bootstrapped standard errors. ^a^indicates 5% significant and ^b^indicates 1% significant

Looking at the change in public drug expenditure following Medicare Part D by income decile, we found the middle and upper income groups among the over 65 s received most of the absolute increase in public drug expenditure between 2003 and 05 and 2006–08 (Additional file 1: Figure S5). The upper income groups also experienced the greatest relative increase in public drug spending. While the lowest two deciles gained 50–70% of additional public expenditures compared to 2003–05, the 7th and 10th deciles more than doubled their 2003–05 benefit level. As a result, the inequality in public drug expenditure became less pro-poor over this period in both relative and absolute terms.

Regression decomposition results from the directly standardised public drug expenditure (Additional file 1: Table S2 and Figure S6) indicate the possible factors (income, gender and ethnicity) associated with the inequality change in public drug expenditure. Across all time periods, higher income was significantly associated with less public drug expenditure (Additional file 1: Table S2). As expected, lower income is related to higher public drug spending, meaning that income makes a positive contribution to the pro-poor inequality in public drug expenditure (Additional file 1: Figure S6). This is also the most important factor that contributes to the public drug expenditure CI, which takes a share that increases from 45 to 70% over time. Prior to Part D, being male and of non-white ethnicity was significantly associated with higher public drug expenditure. These associations reverse following the implementation of Part D with being of Hispanic ethnicity significantly associated with less public drug expenditure compared to whites following Part D. The sign switch is also observed for the contribution of gender and ethnicity to the overall directly standardised partial CI. Income-related inequality in ethnicity was initially contributing pro-poorly before Part D, with non-white individuals being both poorer and having higher publicly funded drug expenditure, but it became a pro-rich component after Part D came into effect, offsetting the overall inequality in public drug expenditure. The income-related inequality in gender had the reverse effect on the public drug expenditure CI.

Our conclusions were robust to most robustness checks, although our point estimates for effect sizes decreased in some of the sensitivity analyses (see Additional file 1: Tables S3-S6 for further details). Standardising expenditure for need using the four medical conditions in addition to self-reported health resulted in small changes in the inequality indices computed compared to standardising for age and self-reported health alone. Re-estimating the DID analysis in this case resulted in a 6–7% reduction in the effect size of Part D on inequality of public expenditure in both relative and absolute terms (but was still statistically significant). There was no sizeable change in effect size of Part D on the GCI of private drug expenditure compared to the base case analysis standardising for need using age and self-reported health alone.

Removing the Medicare population from the under 65 group resulted in slightly stronger relative effects and slightly weaker absolute effects on inequality in public and private expenditures following Part D, but with overall similar magnitude and significance levels. Finally, including imputed premiums for low-income individuals and dual eligibles had very little effect on results when added in as income effect. On the other hand, when imputed premiums were added to public drug expenditures our DID results for public drug expenditure were, as expected, smaller for the CI, although still significant, while our DID estimates for GCI were also smaller and no longer significantly different from zero.

Estimating the medium term impact of Part D by using the 2008–2010 data as the “post Part D” period rather than 2006–2008 resulted in much smaller effect sizes compared to the estimated immediate impact. The DID estimates of CI in public drug expenditure were no longer significantly different from zero. Only the DID estimates of CGI in public and private drug expenditure remained significant at the 5 and 10% levels respectively.

## Discussion

The implementation of Part D significantly increased public drug expenditure while reducing OOP and private drug expenditure. OOP and public drug expenditures were higher among the poor compared to the rich (pro-poor inequality) among Medicare beneficiaries before and after Part D. Our DID analysis suggest that there was no distributional impact of Part D on inequality in OOP expenditure. However, we find Part D led to differential changes in the relative inequality in public drug expenditure between the under 65 s and the over 65 s, with a significant reduction in the degree of pro-poor inequality among Medicare beneficiaries over 65 years of age following its implementation. This means that the rich gained more of the overall increase in public expenditures on prescription drugs from 2003 to 05 to 2006–08 compared to the poor, in both relative and absolute terms. While our point estimates were smaller in some of our robustness checks, the general trends still held true across most sensitivity analyses.

Prior to 2006, public funding for prescription drugs was oriented towards families and individuals with very low income and limited resources, primarily through the Medicaid program. Part D enabled the uptake of public subsidized coverage for prescription drugs among all Medicare beneficiaries, regardless of income. We find that total drug expenditure remained fairly constant over the period 2006–08 and 2009–11 in both the elderly and non-elderly, despite changes in the sources of expenditures, with lower out-of-pocket spending, and higher public spending. There are several possible explanations for this, including the recession in 2008 and 2009, and the lowered unit price of drugs, due to switches to generics and decrease in the price of generics [26, 45]. We also found a significant reduction in the pro-rich inequality of private drug expenditures among the elderly as compared to the near elderly in absolute terms. This indicates an absolute reduction in the amount of spending on prescription drugs through private insurance coverage among the rich as compared to the poor in the over 65 s following Part D. Others have found evidence of substantial crowding-out following the implementation of Part D, with a reduction in other forms of prescription drug coverage as Medicare beneficiaries switch to subsidised drug coverage under Part D plans [12].

Further investigation showed that individuals ranked in the middle and upper income groups gained more public prescription drug assistance in both relative and absolute terms than the poorer income groups as a result of Part D. We also observe important changes over time in the role of gender and ethnicity with respect to public drug spending and the percentage contribution to inequality in public drug expenditure. Notably, we find poorer non-white ethnic groups did not benefit as much from Part D as their richer counterparts. In summary, the policy had a disproportionate effect on the distribution of public drug expenditure.

Several factors could explain our findings. First, many higher income beneficiaries had generous drug coverage prior to Part D, and may have had higher existing use of prescription drugs compared to those gaining coverage under Part D. A review found numerous studies documenting cost-related non-adherence to prescription drugs among lower-income beneficiaries prior to Part D [46]. The implementation of Part D was found to be associated with a small but significant reduction in cost-related non-adherence [47]. Second, co-pays and coverage gaps are still likely to impact on lower income vulnerable individuals through non-adherence of prescription drugs. For example, the near poor who do not qualify for low-income subsidies may be particularly hard hit. Beneficiaries who are dually eligible for Medicaid and Medicare may also represent a vulnerable group. Previously covered under Medicaid, they would have been moved to plans that vary in generosity. Although dual eligibles and LIS-eligible beneficiaries may face no or a very low deductable and monthly premium, their co-pays may be higher than before, and drugs may or may not be included on their plans’ formulary. Finally, it is possible that aside from increased usage compared to lower-income groups, higher income individuals may use newer, more expensive drugs.

There are important limitations to consider. First, we use cross-sectional data from individuals followed for up to two years. Any changes in demographics over time may impact our estimated measures of inequality in drug expenditures. Next, we use real equivalized household income as a measure of welfare, which reflects pre-tax income, since MEPS does not provide publicly available after-tax income for individuals and households [48]. Given a substantial proportion of our over 65 population group are likely to be retired, household income may be an imperfect measure of household welfare. Unfortunately, information on household assets is not publicly available in the MEPS data. Reassuringly, a comparison of health inequality across countries found very little difference in health-related CI when using income or wealth among an older population (aged 50 and over) in the U.S. [49].

Third, we were unable to include Part D premiums. While, cost-sharing arrangements associated with LIS plans are already reflected in drug expenditures, since LIS eligible beneficiaries are exempt from some (if not all) monthly premiums, this exclusion could bias the measured amount of inequality leading us to overestimate the reduction in pro-poor inequality in public expenditure following Part D. Treating premium subsidies for low-income and dual eligible beneficiaries as additional public drug expenditure reduced the estimated effect of Part D in terms of relative inequality, with no significant effect noted in absolute terms. This scenario represents a lower-bound estimate given it assumes the full amount of premiums waived would have been spent on drugs.

It is unclear to what extent errors in the classification of drug expenditures in the 2007 data between private and public may have influenced our results. While we carry out a robustness check excluding the year 2007, the results from this sensitivity analysis reflect a period 3 to 5 years post implementation of Part D, and thus estimates the medium-term impact of the policy change.

Our control group is made up of individuals aged 54 to 63 years old, which may suffer from a potential lack of comparability with the Medicare-eligible population aged 65 years and above. Unfortunately, adults who don’t enrol in Medicare Part D would not be considered an appropriate control group due to selection effects. For example, low income individuals dually eligible for Medicaid and Medicare, and other low-income beneficiaries, were automatically enrolled in Medicare Part D. We do not exclude individuals under 65 years who were also receiving Medicare assistance due to a long-term disability in order to assess the level of inequality in the whole population. Medicare coverage in our non-elderly sample was 6% prior to Medicare Part D. After 2006, less than 5% of individuals in our non-elderly sample were enrolled in Part D. As a result, our estimation of the effect of Part D on income-related inequality using the DID approach may be slightly downward biased. Our robustness check indicated a similar magnitude of effect and significance levels for the period immediately following the implementation of Part D when individuals on Medicare were removed from our under 65 sample.

Although Part D was the major policy that came into effect during the study period, other policy changes during that period may have biased our findings. The effects we note here are specific to the period immediately prior to and following the implementation of Medicare Part D. As the prescription drug benefit changes over time under the Affordable Care Act, with lower enrolee co-payments in the coverage gap (or “doughnut-hole”), the level of OOP costs will likely decrease while plan-related costs will go up. Thus the impact on the inequality of the policy will likely also evolve over time, as we noted in one of our robustness checks. Finally, we did not consider how Part D affected the individual risk of OOP drug expenditure and the differential impact of this across the income distribution [50]; this is an important aspect to consider in future evaluations.

## Conclusions

This analysis illustrates how changes in the level and entitlements to publicly financed health insurance can be evaluated in terms of benefits to low- and high-income groups using well-established income-related health expenditure inequality indices. While many studies have evaluated the level of income-related health inequality within and across countries at different levels of time, it is rare to find a policy change that can allow for an assessment of the impact on the amount of inequality in a population. The Medicare Modernization Act of 2003, through which Part D was established, represents the most significant expansion of public insurance in the U.S. over the past four decades [12]. One of the main goals of Medicare Part D was to lower financial barriers to accessing medications among the elderly, especially among vulnerable groups such as the poor and chronically ill. Others have found that Part D has not adequately protected higher risk beneficiaries [17]. We show that increased public spending on prescription drugs in the years following the implementation of Part D were consumed more by the rich than the poor among those over 65 and as a result, the degree of pro-poor inequality in public drug expenditures fell immediately following the implementation of Part D.

This study demonstrates the importance of evaluating not only the average impact of a policy but also the differing impacts across socioeconomic groups and other important sub groups of the population to determine the winners and losers of any policy. While examining the impact of policy changes on progressivity of taxation has been used widely in empirical analysis (e.g. [51]), there has been much less attention paid to changes in the progressivity of government entitlements following major health policy changes. This analysis highlights the importance of considering the fairness of expanded public insurance programs through the use of well-established health inequality indices, and shows that it is feasible to undertake this task using routinely collected data.

## Additional file

Additional file 1: **Table S1.** Selected years’ CI in drug expenditure. **Figure S1.** Income-related inequality in drug expenditure (CI) from all sources. **Figure S2.** Income-related inequality in drug expenditure (GCI) from all sources. **Figure S3.** Inequality in directly standardised drug expenditure (CI). **Figure S4.** Inequality in indirectly standardised drug expenditure (CI). **Figure S5.** Weighted average public drug expenditure by income decile pre- and post- Medicare Part D (for the over 65s). **Table S2.** Coefficients of directly standardised equations of public drug expenditure. **Figure S6.** Decomposition of directly standardised CI (over 65s). **Table S3.** Difference-in-differences estimation in CI and GCI of drug expenditure. **Table S4.** Difference-in-differences estimation in CI and GCI of drug expenditure. **Table S5.** Difference-in-differences estimation in mean, CI and GCI of drug expenditure. **Table S6.** Difference-in-differences estimation in mean, CI and GCI of drug expenditure. (DOCX 1402 kb)

## Abbreviations

CI: Concentration index
CPI: Consumer Price Index
DID: Difference-in-differences
FPL: Federal Poverty Line
GCI: Generalised concentration index
ICD-9-CM: International Classification of Diseases, Ninth Revision, Clinical Modification
LIS: Low-Income Subsidy
MEPS: Medical Expenditure Panel Survey
OECD: Organisation for Economic Co-operation and Development
OOP: Out-of-pocket
Part D: Medicare prescription drug benefit
PDP: Prescription drug plan
U.S.: United States
