# Supplementary material for: The impact of Medicare part D on income-related inequality in pharmaceutical expenditure
Source: Int J Equity Health. 2019 Apr 16;18:57. doi: 10.1186/s12939-019-0955-9 (PMC6469203; doi:10.1186/s12939-019-0955-9)
Supplement: Supplementary file 1 — Table S1. Selected years’ CI in drug expenditure. Figure S1. Income-related inequality in drug expenditure (CI) from all sources. Figure S2. Income-related inequality in drug expenditure (GCI) from all sources. Figure S3. Inequality in directly standardised drug expenditure (CI). Figure S4. Inequality in indirectly standardised drug expenditure (CI). Figure S5. Weighted average public drug expenditure by income decile pre- and post- Medicare Part D (for the over 65s). Table S2. Coefficients of directly standardised equations of public drug expenditure. Figure S6. Decomposition of directly standardised CI (over 65s). Table S3. Difference-in-differences estimation in CI and GCI of drug expenditure. Table S4. Difference-in-differences estimation in CI and GCI of drug expenditure. Table S5. Difference-in-differences estimation in mean, CI and GCI of drug expenditure. Table S6. Difference-in-differences estimation in mean, CI and GCI of drug expenditure. (DOCX 1402 kb) [file 12939_2019_955_MOESM1_ESM.docx]

**Additional file 1**

**1. Standardisation Methods**

We use both direct and indirect standardisation techniques to standardise individual drug expenditure for need differences using age and self-reported health levels. These methods will not necessarily produce the same results.

**1.1 Direct Standardisation**

We estimate the following equation for each individual $i$:

(1) $E_{i}=\alpha^{D}+\sum_{j} \beta_{j}^{D}x_{ij}+{\gamma_{1}}^{D}I_{i}+{\gamma_{2}}^{D}{male}_{i}+{\gamma_{3}}^{D}{race}_{i}+\varepsilon_{i}^{D}$

where $E_{i}$ is the drug expenditure variable; $x_{ij}$ are the policy non-relevant variables that we standardise for (age group and self-reported health dummies);$I_{i}$ is the individual’s equivalised household income, ${male}_{i}$ is gender, ${race}_{i}$ represents race and ethnicity dummies and $\varepsilon_{i}$ is the unexplained error term.

We then predict the directly standardised drug expenditure using:

(2) $\hat{E}_{i}^{D}=\hat{\alpha}^{D}+\sum_{j} \hat{\beta}_{j}^{D}x_{ij}^{0}+{\hat{\gamma}_{1}}^{D}I_{i}+{\hat{\gamma}_{2}}^{D}{male}_{i}+{\hat{\gamma}_{3}}^{D}{race}_{i}+\hat{\varepsilon}_{i}^{D}$

where $\hat{\alpha}$, $\hat{\beta}_{j}$, $\hat{\gamma}_{k}$ are estimated coefficients and $\hat{\varepsilon_{i}}$ is the residual from the equation above. $x_{ij}^{0}$ is taken for the baseline individual who is defined as one with age in the lowest age group and excellent health. Since the variables in $x_{ij}$ are dummies, we essentially reduce the directly standardised expenditure to:

(3) $\hat{E}_{i}^{D}=\hat{\alpha}^{D}+{\hat{\gamma}_{1}}^{D}I_{i}+{\hat{\gamma}_{2}}^{D}{male}_{i}+{\hat{\gamma}_{3}}^{D}{race}_{i}+\hat{\varepsilon}_{i}^{D}$

This directly standardised expenditure is then used to estimate the partial CI and partial GCI:

(4) ${PCI}^{D}=\frac{\bar{E}^{D}}{\bar{E}}{CI}^{D}$ (5) $PG{CI}^{D}=\bar{E}^{D}{CI}^{D}$

where $\bar{E}^{D}$ is the weighted average of $\hat{E}_{i}^{D}$ and $\bar{E}$ is the weighted average of $E_{i}$ as before. ${CI}^{D}$ is the CI calculated using the standardised expenditure and relative income rank.

We also decompose the directly standardised CI and GCI into the percent contribution from income, gender and ethnicity ([Clarke et al., 2003](#_ENREF_10)).

**1.2 Indirect Standardisation**

For indirect standardisation, we first estimate the following equation for each individual using the same standardisation variables:

(6) $E_{i}=\alpha^{IN}+\sum_{j} \beta_{j}^{IN}x_{ij}+\varepsilon_{i}^{IN}$

The predicted value is then obtained as

(7) $\hat{E}_{i}=\hat{\alpha}^{IN}+\sum_{j} \hat{\beta}_{j}^{IN}x_{ij}$

which is used to calculate the indirectly standardised expenditure:

(8) $\hat{E}_{i}^{IN}=E_{i}-\hat{E}_{i}+\bar{E}$

The partial CI and GCI calculated using indirectly standardised expenditure are:

(9) ${PCI}^{IN}={CI}^{IN}$ (10) $G{PCI}^{IN}=\bar{E}^{IN}{CI}^{IN}$

**2. Sample and Analysis**

**2.1 Difference-in-differences estimation**

Another way to present the DID model is as follows:

(11) Y= β0 + β1*[Time] + β2*[Intervention] + β3*[Time*Intervention] + β4*[Covariates]+ε

Where y is the outcome variable of interest, in our case 3 separate outcomes: mean drug expenditure, CI of drug expenditure, and GCI of drug expenditure.

As covariates are not included in the model, the model reduces to:

(12) Y= β0 + β1*[Time] + β2*[Intervention] + β3*[Time*Intervention] + ε

Where

β0 represents the average value of the outcome variable for the control group in the baseline (pre-intervention) period,

β1 represents the time trend for the outcome variable in the control group,

β2 represents the difference in average values of the outcome variable for the treatment group compared to the control group in the baseline (pre-intervention) period,

β3 represents the difference in changes over time, or the DID estimator, $\delta$, as specified in the first model specification.

**2.2 MEPS Data**

The family unit was identified using the MEPS definition (defined similar to the Current Population Survey definition except that it includes foster children and unmarried partners living together who consider themselves a family unit).

**3. Results**

Table S1: Selected years’ CI in drug expenditure

|  | 2003-2005 | | 2006-2008 | |
| --- | --- | --- | --- | --- |
|  | Under 65 | Over 65 | Under 65 | Over 65 |
| Total | –0.082*** | –0.046*** | –0.046** | –0.016 |
|  | (–5.933) | (–4.524) | (–2.307) | (–1.478) |
| OOP | –0.081*** | –0.027*** | –0.051 | –0.002 |
|  | (–3.870) | (–2.853) | (–1.468) | (–0.218) |
| Public | –0.558*** | –0.219*** | –0.543*** | –0.088*** |
|  | (–16.930) | (–13.253) | (–27.228) | (–7.036) |
| Private | 0.094*** | 0.114*** | 0.163*** | 0.223*** |
|  | (5.428) | (2.627) | (7.541) | (7.257) |

*Note: T-statistics in brackets. * indicates 10% significant, ** indicates 5% significant and *** indicates 1% significant.*

Figure S1: Income-related inequality in drug expenditure (CI) from all sources

*Notes: Weighted statistics. The dotted line indicates when Medicare Part D was implemented.*

Figure S2: Income-related inequality in drug expenditure (GCI) from all sources

Figure S3: Inequality in directly standardised drug expenditure (CI)

Figure S4: Inequality in indirectly standardised drug expenditure (CI)

Figure S5: Weighted average public drug expenditure by income decile pre- and post- Medicare Part D (for the over 65s)


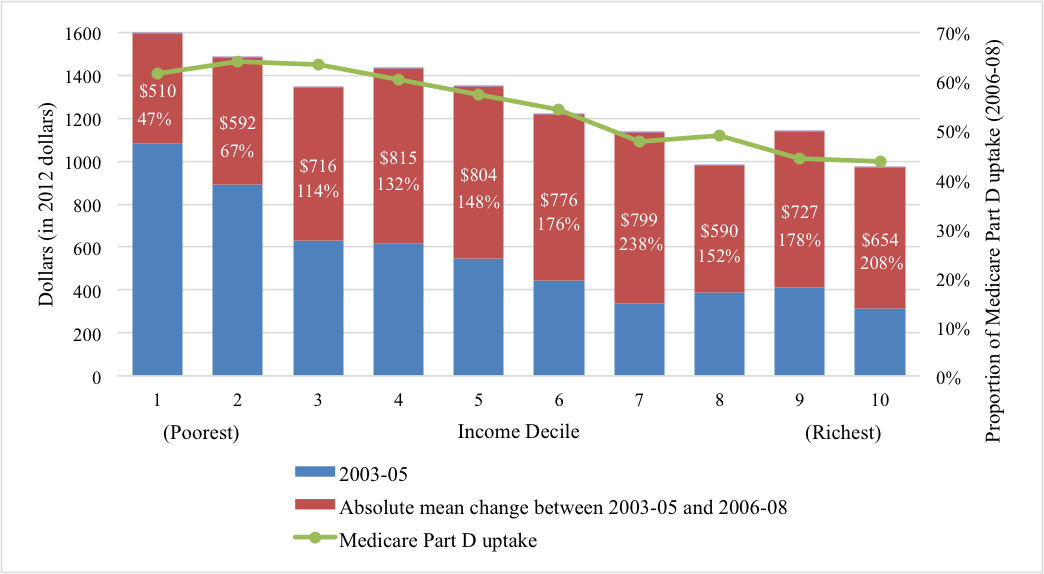


*Notes: Household Equivalised income deciles are arranged from poorest to richest. Numbers on the change bars represent the percentage of that change relative to the expense level in 2003-2005 and the absolute value of the increase in dollars.*

Table S2: Coefficients of directly standardised equations of public drug expenditure

| Public drug expenditure | 1997-99 | 2000-02 | 2003-05 | 2006-08 | 2009-11 |
| --- | --- | --- | --- | --- | --- |
| Income | –0.873*** | –1.905*** | –3.293*** | –1.618** | –3.067*** |
|  | (–4.18) | (–6.61) | (–6.83) | (–2.40) | (–3.89) |
| Male | 30.5* | 129.1*** | 133.7*** | –155.8*** | –69.7 |
|  | (1.82) | (4.89) | (4.15) | (–2.99) | (–1.15) |
| Ethnicity |  |  |  |  |  |
| Black | 132.4*** | 129.3*** | 240.3*** | –87.7 | –72.0 |
|  | (4.10) | (2.89) | (3.67) | (–1.20) | (–0.87) |
| Hispanic | 167.7*** | 248.5*** | 156.6*** | –94.1 | –265.9*** |
|  | (3.81) | (5.14) | (2.87) | (–0.90) | (–2.66) |
| Other | 76.5 | 263.8*** | 202.1*** | 87.9 | –136.6 |
|  | (0.86) | (3.25) | (2.71) | (0.74) | (–1.12) |
| Constant | 91.9*** | 68.8** | 215.2*** | 620.1*** | 512.7*** |
|  | (4.29) | (2.55) | (4.75) | (8.02) | (6.32) |
|  |  |  |  |  |  |
| R^2^ | 0.041 | 0.044 | 0.047 | 0.053 | 0.047 |
| Observations | 9353 | 10912 | 11050 | 10780 | 11552 |

*Notes: T-statistics in brackets. * indicates 10% significant, ** indicates 5% significant and *** indicates 1% significant. Income refers to household equivalised income. The reference group for Ethnicity is White. Self-reported health and age group dummies are not reported.*

Figure S6: Decomposition of directly standardised CI (over 65s)

***Robustness checks***

**Extra standardisation for health conditions**

DID results for public and private expenditure when standardising expenditure for four medical conditions in addition to self-reported health are shown below.

Table S3: Difference-in-differences estimation in CI and GCI of drug expenditure

|  | DID in drug expenditure CI | | DID in drug expenditure GCI | |
| --- | --- | --- | --- | --- |
|  | Standardisation | | Standardisation | |
|  | Direct | Indirect | Direct | Indirect |
| Robustness check results: Standardise for need using age group, self-reported health and 4 additional diagnoses | | | | |
| Public | 0.099** | 0.099** | 64.144*** | 62.104*** |
|  | (2.229) | (2.425) | (2.914) | (3.044) |
| Private | 0.026 | 0.021 | -74.095** | -67.595** |
|  | (0.476) | (0.436) | (-2.290) | (-2.337) |
| Original results: Standardise for need using age group and self-reported health | | | | |
| Public | 0.107** | 0.107** | 68.255*** | 66.254*** |
|  | (2.375) | (2.573) | (2.999) | (3.121) |
| Private | 0.030 | 0.027 | -74.460** | -67.659** |
|  | (0.536) | (0.527) | (-2.177) | (-2.192) |

*Notes: DID estimation over the period 2003-05 and 2006-08. T-statistics, in brackets, based on bootstrapped standard errors. * indicates 10% significant, ** indicates 5% significant and *** indicates 1% significant.*

**Estimating the medium term impact by using 2008-2010 as “post” Part D period**

DID results for inequality in public and private drug expenditure using 2008-2010 as the post-implementation period are shown below.

Table S4: Difference-in-differences estimation in CI and GCI of drug expenditure

|  | DID in drug expenditure CI | | | DID in drug expenditure GCI | | |
| --- | --- | --- | --- | --- | --- | --- |
|  | Standardisation | | | Standardisation | | |
|  | None | Direct | Indirect | None | Direct | Indirect |
| Robustness check results: DID estimation over the period 2003-05 and 2008-10 | | | | | | |
| Public | 0.039 | 0.064 | 0.068 | 27.545 | 53.939** | 53.103** |
|  | (0.892) | (1.394) | (1.592) | (0.970) | (2.099) | (2.215) |
| Private | 0.005 | 0.003 | -0.003 | -37.403 | -48.514 | -46.883* |
|  | (0.089) | (0.065) | (-0.060) | (-1.203) | (-1.553) | (-1.652) |
| Original results: DID estimation over the period 2003-05 and 2006-08 | | | | | | |
| Public | 0.117*** | 0.107** | 0.107** | 50.413** | 68.255*** | 66.254*** |
|  | (2.810) | (2.375) | (2.573) | (2.006) | (2.999) | (3.121) |
| Private | 0.040 | 0.030 | 0.027 | -48.646 | -74.460** | -67.659** |
|  | (0.667) | (0.536) | (0.527) | (-1.525) | (-2.177) | (-2.192) |

*Notes: T-statistics, in brackets, based on bootstrapped standard errors. * indicates 10% significant, ** indicates 5% significant and *** indicates 1% significant.*

**Removing <65s with Medicare from the control group**

Results from the DID estimation carried out after removing the Medicare population from the near-elderly (under 65) group are shown below.

Table S5: Difference-in-differences estimation in mean, CI and GCI of drug expenditure

|  | DID in mean drug expenditure | DID in drug expenditure CI | | | | | DID in drug expenditure GCI | | | | |
| --- | --- | --- | --- | --- | --- | --- | --- | --- | --- | --- | --- |
|  |  | Standardisation | | | | | Standardisation | | | | |
|  |  | None | Direct | | Indirect | | None | Direct | | Indirect | |
| Total | 79.7 | -0.003 | | -0.006 | | -0.004 | 14.459 | | 12.868 | | 13.642 |
|  | (0.892) | (-0.102) | | (-0.191) | | (-0.149) | (0.291) | | (0.256) | | (0.293) |
| OOP | -337.4*** | 0.044 | | 0.051 | | 0.047 | 35.470 | | 26.298 | | 23.845 |
|  | (-9.184) | (0.945) | | (1.136) | | (1.139) | (1.409) | | (1.071) | | (1.040) |
| Public | 684.8*** | 0.160*** | | 0.156*** | | 0.153*** | 25.969 | | 58.575*** | | 56.972*** |
|  | (16.963) | (3.660) | | (3.415) | | (3.601) | (1.125) | | (2.778) | | (2.886) |
| Private | -267.7*** | 0.042 | | 0.032 | | 0.027 | -46.980 | | -72.005** | | -67.174** |
|  | (-4.196) | (0.717) | | (0.584) | | (0.537) | (-1.478) | | (-2.068) | | (-2.114) |

*Notes: DID estimation over the period 2003-05 and 2006-08. T-statistics, in brackets, based on bootstrapped standard errors. * indicates 10% significant, ** indicates 5% significant and *** indicates 1% significant.*

**Imputing premiums for low-income and dual eligible beneficiaries**

Adding in premiums as income (income effect) had very little influence on results. (Results not shown). Results are shown below for the DID estimation carried out when adding premiums to public drug expenditures.

Table S6: Difference-in-differences estimation in mean, CI and GCI of drug expenditure

|  | DID in mean drug expenditure | DID in drug expenditure CI | | | | | DID in drug expenditure GCI | | | | |
| --- | --- | --- | --- | --- | --- | --- | --- | --- | --- | --- | --- |
|  |  | Standardisation | | | | | Standardisation | | | | |
|  |  | None | Direct | | Indirect | | None | Direct | | Indirect | |
| Total | 14027 | -0.023 | | -0.027 | | -0.023 | -34.464 | | -36.877 | | -30.822 |
|  | (1.524) | (-0.858) | | (-0.965) | | (-0.901) | (-0.684) | | (-0.721) | | (-0.661) |
| OOP | -285.7*** | 0.005 | | 0.010 | | 0.011 | 3.742 | | 7.068 | | 6.023 |
|  | (-7.425) | (-0.118) | | (0.251) | | (0.277) | (0.148) | | (0.289) | | (0.267) |
| Public | 685.3*** | 0.091** | | 0.080* | | 0.081** | 10.445 | | 30.521 | | 30.820 |
|  | (15.737) | (2.166) | | (1.778) | | (1.969) | (0.414) | | (1.334) | | (1.444) |
| Private | -259.4*** | 0.040 | | 0.030 | | 0.027 | -48.65 | | -74.465** | | -67.665** |
|  | (-4.102) | (0.685) | | (0.537) | | (0.529) | (-1.540) | | (-2.166) | | (-2.180) |

*Notes: DID estimation over the period 2003-05 and 2006-08. T-statistics, in brackets, based on bootstrapped standard errors. * indicates 10% significant, ** indicates 5% significant and *** indicates 1% significant.*
